# A comparison between cisatracurium and rocuronium-induced neuromuscular block on laryngeal electromyography recovery after neostigmine reversal in a porcine model

**DOI:** 10.3389/fendo.2022.875597

**Published:** 2022-08-08

**Authors:** I-Cheng Lu, Hao Tan, Sheng-Hua Wu, Tzu-Yen Huang, Hsin-Yi Tseng, Jia-Joanna Wang, Gianlorenzo Dionigi, Young Jun Chai, Feng-Yu Chiang, Pi-Ying Chang, Che-Wei Wu

**Affiliations:** ^1^ Department of Anesthesiology, Kaohsiung Municipal Siaogang Hospital, Kaohsiung Medical University Hospital, Kaohsiung Medical University, Kaohsiung, Taiwan; ^2^ Faculty of Medicine, College of Medicine, Kaohsiung Medical University, Kaohsiung, Taiwan; ^3^ Department of Anesthesiology, Kaohsiung Municipal Ta-Tung Hospital, Kaohsiung Medical University Hospital, Kaohsiung Medical University, Kaohsiung, Taiwan; ^4^ Department of Otorhinolaryngology, Kaohsiung Medical University Hospital, Kaohsiung Medical University, Kaohsiung, Taiwan; ^5^ Division of General Surgery, Endocrine Surgery Section, Istituto Auxologico Italiano, Istituto di ricovero e cura a carattere scientifico, Milan, Italy; ^6^ Department of Pathophysiology and Transplantation, University of Milan, Milan, Italy; ^7^ Department of Surgery, Seoul National University College of Medicine, Seoul Metropolitan Government—Seoul National University Boramae Medical Center, Transdisciplinary Department of Medicine & Advanced Technology, Seoul National University Hospital, Seoul, South Korea; ^8^ Department of Otolaryngology, E-Da Hospital, I-Shou University, Kaohsiung, Taiwan

**Keywords:** intraoperative neuromonitoring (IONM), thyroid surgery, laryngeal electromyography (EMG), neostigmine, neuromuscular blocking agent (NMBA), recurrent laryngeal nerve (RLN), voice, precision medicine

## Abstract

**Background:**

Inducing and reversing neuromuscular block is essential to a positive outcome of thyroid surgery, with intraoperative neuromonitoring (IONM) being used to decrease recurrent and superior laryngeal nerve injuries and improve vocal outcome. Neostigmine is a non-specific broad-spectrum and inexpensive reversal agent for neuromuscular blocking agents (NMBAs). The aim of this porcine study was to explore the effect of neostigmine on electromyography (EMG) signal recovery profile following the commonly used NMBAs, cisatracurium and rocuronium.

**Methods:**

Twelve piglets were allocated into two groups with six piglets in each group. When stable baseline EMG signals were obtained, a neuromuscular block was induced by intravenous cisatracurium 0.2 mg/kg (group C) or rocuronium 0.6 mg/kg (group R) for each piglet. We compared laryngeal EMG tracing with spontaneous recovery (control) and neostigmine (0.04 mg/kg) reversal for each group. The time course of real-time laryngeal EMG signals was observed for 30 min from NMBA injection. Effects of neostigmine on EMG signal were assessed at 50% EMG recovery and by the maximum neuromuscular block recovery degree from the baseline value.

**Results:**

Neostigmine shortened the recovery time to 50% EMG amplitude in both group C (16.5 [2.5] vs. 29.0 [2.0] min, *P*<0.01) and group R (16.5[2.5] vs. 26.5 [1.5] min, *P*<0.05) compared to spontaneous recovery, respectively. Neostigmine reversal also enhanced the maximum degree of EMG amplitude recovery in both group C (83.6 [5.1] vs. 47.2 [6.1] %, *P*<0.01) and group R (85.6 [18.2]vs. 57.1 [6.3] %, *P*<0.05) compared to spontaneous recovery, respectively. The reversal effect of neostigmine did not differ significantly between cisatracurium and rocuronium.

**Conclusions:**

This porcine model demonstrated that neostigmine provides an adequate and timely IONM signal suppressed by both cisatracurium and rocuronium. These results can potentially expand the options for precision neuromuscular block management during IONM to improve vocal outcomes in thyroid surgery patients.

## Introduction

For decades, thyroid surgeons have been improving vocal outcomes after thyroid surgery through the use of intraoperative neuromonitoring (IONM). IONM is used to prevent or predict recurrent laryngeal nerve (RLN) injury as it provides RLN identification, variant nerve detection, and nerve injury differentiation (1–9). A key factor to ensure functional IONM is the correct management of the degree of neuromuscular block (NMB). An ideal NMB degree is complete to profound for tracheal intubation during anesthesia induction, and moderate to shallow for electromyography (EMG) signals during IONM (10, 11).

With respect to NMB management for IONM during thyroid surgery, most trials have investigated a combination of rocuronium and sugammadex. Rocuronium protocols for IONM have been established as: standard dose, one-effective dose, or in combination with sugammadex (12–14). Sugammadex is produced as a specific reversal agent for aminosteroidal NMBAs (15, 16). Sugammadex can be effectively titrated to restore EMG signals suppressed by rocuronium-induced NMB from deep to shallow block degrees (17, 18). However, this combination is not available everywhere and very expensive. Neostigmine is a broad-spectrum and inexpensive reversal agent to non-depolarizing NMBAs. Neostigmine may be an alternative option for IONM. Recently, neostigmine has been successfully used to facilitate IONM under rocuronium-induced NMB (19).

Cisatracurium is the most widely used non-depolarizing NMBA with an isoquinoline structure and has two major advantages in clinical applications. First, it has a stable hemodynamic profile. Cisatracurium does not induce histamine release as do other isoquinoline agents (ex. atracurium), which may cause hypotension. Second, it has a favorable metabolism profile. The metabolism is *via* Hoffman elimination, which is dependent on body temperature and independent of liver or renal function. Hence, cisatracurium is also feasible for patients with organ failure or the elderly (20, 21). Only a limited of studies have investigated the application of cisatracurium in IONM during thyroid surgery (22).

To the best of our knowledge, there are no studies comparing cisatracurium with rocuronium during IONM. The purpose of this study was to explore the effect of neostigmine on EMG recovery profile in pigs receiving either cisatracurium or rocuronium during thyroid surgery. It was hypothesized that neostigmine may be effective for reversing both cisatracurium- and rocuronium-induced neuromuscular block and consequently allowing functional IONM. We compared neostigmine reversal to spontaneous recovery at an institutional intubation dose of cisatracurium (0.2 mg/kg) and rocuronium (0.6 mg/kg).

## Methods

### Animal preparations and anesthesia

The prospective animal study was approved by the Institutional Animal Care and Use Committee of Kaohsiung Medical University (protocol No: 109121). Twelve male piglets aged 3–4 months and weighing 18–22 kg were obtained from the Laboratory Animal Center of Kaohsiung Medical University. All porcine experiments were performed according to institutional guidelines that strictly followed international regulations and national policy. Electromyography of the porcine experiment, consisting of threshold, latency, amplitude, and evoked potentials, were comparable to human data in established animal models (23–25).

All piglets were fasted for 8 h, but allowed water 2 h before the experiments. Premedication included intramuscular azaperone (4 mg/kg) and zoletil (5 mg/kg) 30 min before general anesthesia. Anesthesia induction was performed in the ventrodorsal position by inhalation of 1%–2% isoflurane *via* a hollow plastic bottle connected to an anesthetic machine. After cannulation of a 24-gauge catheter into the peripheral vein on the ear, each piglet was intubated with an EMG endotracheal tube with an inner diameter of 6.0 mm (Medtronic, Jacksonville, FL, USA) under direct laryngoscopy. General anesthesia was maintained with isoflurane 1%–1.3%, and the piglets were control-ventilated with a minute volume of 2–3 L/min. Standard physiological monitoring (electrocardiography, pulse oximetry, noninvasive blood pressure, and capnography) was recorded by a Vista 120 monitor (Drager, Lubeck, Germany) until the experiment ended.

### Surgical procedures and neural monitoring setup

After skin disinfection in the dorsoventral position, a transverse collar incision was made to expose the neck and larynx of each piglet. The strap muscles were removed to reveal the trachea and target nerves (vagus nerve and RLN). Both the vagus nerve and RLN were identified and dissected free from the overlying soft tissue and fascia. The subplatysmal flap was raised cranially from the clavicle to the hyoid bone. Monopolar and bipolar electrocautery were used to facilitate dissection and hemostasis.

To accurately observe real-time EMG changes, continuous intraoperative neuromonitoring (C-IONM) *via* vagus nerve stimulation using an Automatic Periodic Stimulating (APS, Medtronic) accessory was applied (26) to provide seamless monitoring of the functional status along the entire vagus–RLN axis in real time. Stimulation frequency and current for C-IONM was set once per second (1Hz) at 1 mA. After connecting the APS electrode with the Nerve Integrity Monitor system (NIM-Response 3.0, Medtronic), baselines for the latency and amplitude of the evoked response were calibrated automatically. The monitor was set with a response threshold of 100 μV, stimulation rejection artifact at 2.6 ms, and a rectangular pulse negative stimulus of 100 μs duration. Continuous EMG tracings were recorded and analyzed by an NIM-Response 3.0 System (Medtronic) during all animal experiments to continuously evaluate real-time RLN function (27, 28).

### Outcome analysis

**Figure 1:**
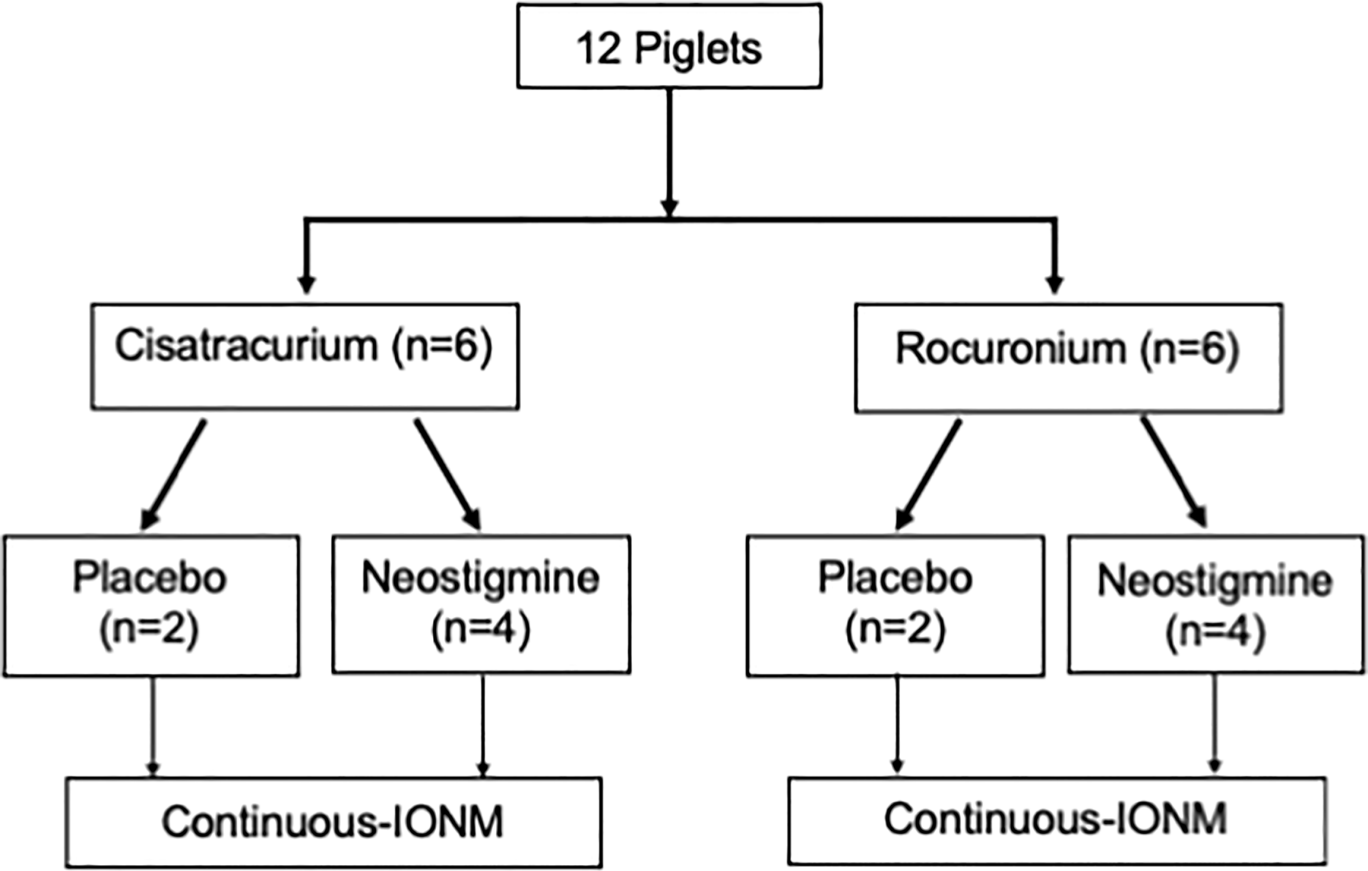
Study Flowchart Of 12 enrolled piglets, eight were reversed by neostigmine (0.04 mg/kg) and four underwent spontaneous recovery with saline (placebo). IONM, intraoperative neuromonitoring.

Twelve piglets were allocated into group C or group R according to different neuromuscular blocking agents with six piglets in each group. The experimental flowchart (**Figure 1**) shows both placebo control and neostigmine reversal pigs within each group. When stable EMG signals were obtained, a neuromuscular block was induced by cisatracurium (0.2 mg/kg) or rocuronium (0.6 mg/kg) for group C and group R, respectively. After a 10-min interval, an intravenous bolus of neostigmine (0.04 mg/kg) was given to reverse NMB in four piglets in both groups. In the remaining two piglets in each group, saline was injected as a placebo to observe spontaneous recovery of EMG signal after cisatracurium or rocuronium. Laryngeal EMG signals were continuously recorded during a 30-min interval from administration of cisatracurium or rocuronium. The main outcomes of neostigmine were assessed by time to a 50% recovery of baseline EMG amplitude and maximum recovery of EMG amplitude within a 30-min interval compared to the baseline value. The secondary outcome was adverse cardiovascular events caused by neostigmine. A 20% decrease in heart rate or blood pressure was defined as bradycardia or hypotension. If bradycardia or hypotension occurred, it was treated with atropine or ephedrine.

### Statistical analysis

This study is a preliminary study on a porcine model and estimation of sample size has not been performed. Assuming a non-normal distribution of the data due to small sample size, non-parametric tests were used. Continuous data are presented as median and interquartile range [IQR] values and nominal data are presented as no (%). Statistical analysis of continuous variables without normal distribution between groups was compared using the Mann–Whitney U-test. All statistical tests were two-tailed. Categorical nominal variables were analyzed using Fisher’s exact test. Statistical significance was set at *P* < 0.05.

## Results

**Figure 2:**
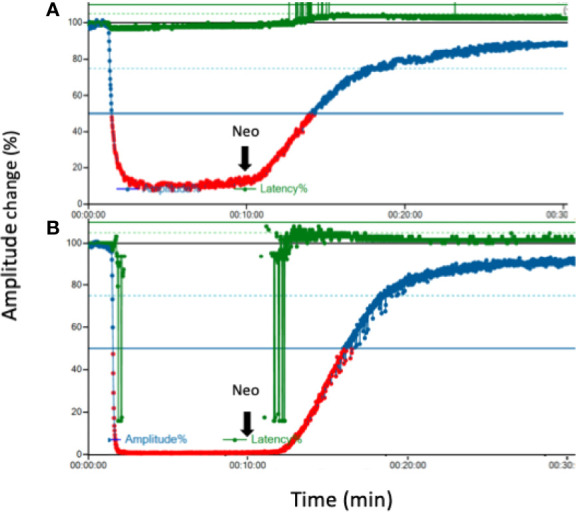
Effect of neostigmine on EMG tracing after neuromuscular block. **(A)** Typical tracing after cisatracurium (0.2 mg/kg) for neuromuscular block **(B)**. Typical tracing after rocuronium (0.6 mg/kg) for neuromuscular block. Ten minutes later, neostigmine (0.04 mg/kg) was injected for each group. An overall observation time was 30 min in each experiment. EMG, electromyography; Neo, neostigmine.

In all 12 piglets, EMG amplitude was undetectable (<100 µV) immediately after injection of cisatracurium (0.2 mg/kg) or rocuronium (0.6 mg/kg) (**Figure 2**). **Figure 2** shows the effect of neostigmine reversal on typical EMG tracing after cisatracurium- and rocuronium-induced neuromuscular block. Neostigmine significantly shortened recovery time to 50% EMG amplitude in piglets receiving both cisatracurium and rocuronium compared to spontaneous recovery (**Table 1** **)**. Recovery time did not differ significantly between cisatracurium and rocuronium either by spontaneous recovery or neostigmine reversal (both *P*>0.05). Neostigmine reversal enhanced the maximum degree of EMG amplitude recovery in both group C and group R compared to spontaneous recovery, respectively (**Table 2**). Maximum recovery degree did not differ significantly between cisatracurium and rocuronium either by spontaneous recovery or neostigmine reversal (both *P*>0.05).

**Table 1:** Recovery time to 50% EMG amplitude.

	Spontaneous recovery (n = 2)	Neostigmine reversal (n = 4)	*P*-value
Group C (min)	29.0 [2.0]	16.5 [2.5]	<0.01
Group R (min)	26.5 [1.5]	16.5 [2.5]	<0.05

EMG, electromyography; Group C received cisatracurium (0.2mg/kg), Group R received rocuronium (0.6 mg/kg). Data are presented as median [IQR].

**Table 2:** Maximum EMG amplitude recovery.

	Spontaneous recovery (n = 2)	Neostigmine reversal (n = 4)	*P*-value
Group C (%)	47.2 [6.1]	83.6 [5.1]	<0.01
Group R (%)	57.1 [6.3]	85.6 [18.2]	<0.05

EMG, electromyography; Group C received cisatracurium (0.2mg/kg), Group R received rocuronium (0.6mg/kg). Data are presented as median [IQR].

**Figure 3:**
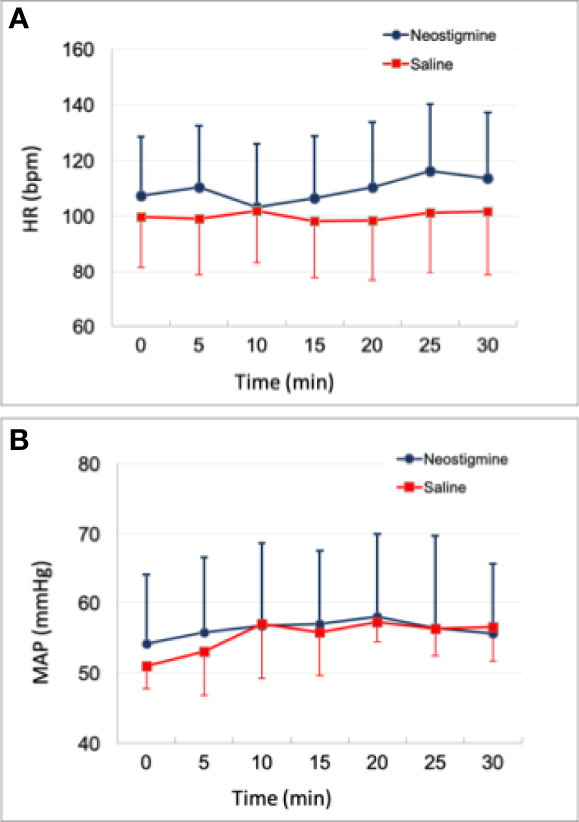
Time course of hemodynamic status. **(A)** Heart rate change **(B)** mean arterial pressure change. Either neostigmine or saline was given to each piglet at 10 min. There was no significant difference between neostigmine (n=8) and saline (n=4) in both heart rate and blood pressure (all *P >*0.05). HR = heart rate; MAP = mean arterial pressure.

**Figure 3** shows the hemodynamic status of all 12 piglets within 30 min. Eight piglets received neostigmine and four piglets received saline at 10 min. In the eight piglets that received neostigmine, heart rate and mean arterial pressure remained unchanged, compared to the four piglets that received saline. There was only a minimal decrease in heart rate (*P*>0.05) when neostigmine was administrated at 10 minute (**Figure 3**). None of animals were given atropine or a vasopressor for bradycardia or hypotension.

## Discussion

The porcine model in this study demonstrated that neostigmine shortened recovery time and enhanced recovery degree of EMG amplitude after cisatracurium- or rocuronium-induced NMB. Recovery time to 50% EMG amplitude after neostigmine administration in group C and group R was near 16.5 min respectively. Maximum recovery degree of EMG amplitude in group C and R was near 86% respectively. Our previous porcine model with sugammadex (0.5 mg/kg) reported a 50% recovery time of 16.8±1.9 min and a maximum recovery degree of 86.8±10.4% (18). In the current study, we confirmed that Neostigmine 0.04 mg/kg provided a similar EMG recovery profile to a low dose of sugammadex. This may explain the recent clinical finding by Oh et al. (19) showing the feasibility of neostigmine reversal of rocuronium in monitored thyroid surgery.

To the best of our knowledge, this study is the first model comparing the reversal effect of neostigmine between cisatracurium and rocuronium for IONM. We demonstrated that neostigmine provided an adequate and timely IONM signal that was suppressed by both cisatracurium and rocuronium. The results expand the options of precision neuromuscular block management during IONM, especially for patients with organ failure or extreme old age. In those population, the use of cisatracurium is more favorable than rocuronium, but cisatracurium-induced NMB is not possible to be reversed by sugammadex. **Figure 2** depicted that cisatracurium 0.2 mg/kg did not completely abolish laryngeal EMG. This occurrence may be explained by suboptimal storage of cisatracurium. Cisatracurium should be refrigerated between 2~8°C strictly to preserve the potency. We lacked refrigerator during transport and in the laboratory to ensure the standard temperature. This might lead to partial loss of predicted potency.

Though several NMB reversal regimens have been suggested, there is still a lack of consensus on NMB management for IONM during thyroid surgery. The management of NMB consists of two aspects: neuromuscular monitoring and NMB reversal. First, neuromuscular monitoring provides a guide to reversal agents based on the degree of NMB. NMB degree during IONM can be quantitatively obtained *via* stimulation on the adductor pollicis muscle with train-of-four (**TOF**), post-tetanic counts mode (11, 29). However, neuromuscular monitoring is not always available even at medical centers in developed countries. Therefore, investigations regarding a universal NMB reversal regimen are valuable for clinical practice. Second, NMB reversal for IONM was aimed at a high-quality EMG signal rather than complete recovery of neuromuscular function. Many studies recount the combination of rocuronium and sugammadex for successful IONM during thyroid surgery (14, 17, 18, 30–32). There is growing evidence that a low dose of sugammadex provides high IONM quality without complete NMB reversal. In two studies investigating 0.5, 1, and 2 mg/kg of sugammadex, it was demonstrated that all doses provided comparable EMG signals and the lower doses (0.5 or 1 mg/kg) were associated with less unwanted movements such as bucking (17, 18).

Since merely partial reversal of NMB is sufficient for IONM, it may be possible to use the non-specific reversal agent neostigmine as an alternative to the selective binding reversal agent sugammadex (19). Neostigmine is also a broad-spectrum reversal agent for all non-depolarizing NMBAs. It may be feasible for both cisatracurium (isoquinoline structure) and rocuronium (aminosteroid structure).

A recent report using neostigmine (2 mg) obtained a sufficient EMG signal with less bucking events in 4% (2/50) of patients than low dose sugammadex (13.7%; 7/51) (17, 19). For this experienced team, time from neostigmine to skin incision and initial EMG signal of the vagus nerve (V1) was 7.7±3.2 and 26.1± 5.5 min, respectively. The longer the waiting period, the higher the EMG amplitude. Hence, the time sequence of this neostigmine protocol is feasible for most thyroid surgery.

Cisatracurium is the commonly used NMBA for general anesthesia but is rarely discussed in association with IONM during thyroid surgery. Most trials focus on the rocuronium-sugammadex regimen because of its effectiveness and stability for successful IONM. Because neostigmine has been shown to be feasible for rocuronium-induced NMB during IONM (19), it should be also feasible for cisatracurium. We compared neostigmine reversal between cisatracurium and rocuronium in a porcine model and found comparable IONM outcomes in recovery time and degree. Thus, we established the feasibility of neostigmine for IONM in thyroid surgeries with two commonly used NMBAs in a porcine model. Further clinical trials are needed to compare cisatracurium-neostigmine between published regimens in clinical practice.

The cisatracurium-neostigmine combination possesses several benefits. Firstly, cisatracurium is well-tolerated in most patients even those in extreme old age or with critical illness (20, 21, 33). Cisatracurium does not induce histamine release and is metabolized by Hoffman elimination which bypasses the liver and kidneys.

There is also an option of using short-acting NMBAs, such as suxamethonium without using neostigmine. This regimen may cause possible disadvantages (e.g., unwanted movements after short term NMB and suxamethonium-induced adverse events). Those disadvantages could be avoided with cisatracurium regimen. Secondly, neostigmine induces adequate EMG amplitude with much less unwanted movement compared with sugammadex. An initial high EMG amplitude is important to identify potential signal reduction (34). In clinical IONM practice, an EMG amplitude > 500 μV is considered as adequate enough for initial vagal stimulation (35). Neostigmine acts as a cholinesterase inhibitor which was very different from sugammadex as a selective relaxant binding agent. Therefore, neostigmine is not as effective as sugammadex to reverse muscle relaxation. Lastly, the cost of cisatracurium-neostigmine is much lower than that of rocuronium-sugammadex. The high price of sugammadex limits its popularity and availability in many healthcare systems (36, 37). Hence, cisatracurium-neostigmine has great potential for IONM because of its effectiveness and accessibility. Recently, cisatracurium titration with spontaneous recovery has been used to facilitate IONM during thyroid surgery (22, 38). A superior IONM outcome of cisatracurium with neostigmine reversal is expected in clinical practice.

There were several limitations in this study. First, this was a prospective animal study with a small sample size. Within each group, four animals were used to test cisatracurium or rocuronium, and saline was used as a placebo in only two animals. The statistical validity of this study is greatly reduced by this. The results of statistical analyses conducted by comparing 2 groups of 2-4 elements each cannot be considered reliable, regardless of whether p is significant or not. However, we found a good reproducibility between experimental animals. The EMG recovery profile was similar in all piglets. The variation between recovery time and degree was relatively small. Secondly, a comparison between neostigmine and sugammadex was not performed in this study. This was because the feasibility of a sugammadex dose from 0.5 to 4 mg/kg has been established in a previous porcine IONM model (14, 18). Our aim was to investigate the feasibility of neostigmine for IONM under different NMBAs. Furthermore, we did not observe significant change of either hemodynamic nor respiratory status due to neostigmine alone in porcine model. However, it should be cautious that cholinesterase inhibitor may lead to not only bradycardia but also more secretion causing fatal problems during mechanical ventilation or postoperatively. Thirdly, it lacks quantitative neuromuscular transmission monitoring such as train-of-four (TOF) to compare the IONM data. We did not use TOF monitor in this study because it is difficult to set up in the porcine model and it will be easy to apply in further clinical study. Finally, caution should be taken in the interpretation and application of the current results. None of the animals experienced significant bradycardia when neostigmine was administered without anticholinergics. Moreover, results of healthy animals undergoing experimental protocols may not be representative of humans with various systemic diseases.

## Conclusions

Neostigmine allowed adequate and timely IONM signal suppression by both cisatracurium and rocuronium in a porcine model. Our results may expand the options of precision NMB management during IONM to improve vocal outcomes in thyroid surgery patients. The EMG recovery profile of cisatracurium-neostigmine or rocuronium-neostigmine was feasible for IONM during thyroid surgery. Although neostigmine (0.04 mg/kg) did not cause significant cardiovascular adverse events in animals, caution should be used when implementing this protocol in a clinical setting.

## Data availability statement

The raw data supporting the conclusions of this article will be made available by the authors, without undue reservation.

## Ethics statement

The animal study was reviewed and approved by Institutional Animal Care and Use Committee of Kaohsiung Medical University.

## Author contributions

C-WW had full access to all the data in the study and takes responsibility for the integrity of the data and the accuracy of the data analysis. P-YC and C-WW contributed equally to this work. Concept and design: I-CL, C-WW, Acquisition, analysis, or interpretation of data: T-YH, H-YT, J-JW. Drafting of the manuscript: I-CL, P-YC, C-WW Critical revision of the manuscript and final approval: All authors Statistical analysis: I-CL, HT Obtained funding: I-CL, T-YH, C-WW Administrative, technical, or material support: S-HW, GD, YC, F-YC Supervision: P-YC, C-WW. All authors contributed to the article and approved the submitted version.

## Funding

This study was supported by grants from the Kaohsiung Medical University Hospital (KMUH110-OR52), the Kaohsiung Municipal Siaogang Hospital (H-109-001, Kmhk-110-08), the Ministry of Science and Technology, Taiwan (MOST 109-2314-B-037-059, MOST 110-2314-B-037-104 -MY2).

## Acknowledgments

The authors express gratitude to Hsiu-Ya Chen, Jung-Yen Hu (nurse anesthetist, Department of Anesthesiology, KMUH, KMU), Hui-Chun Chen (clinical nurse specialist, Department of Nursing, KMUH, KMU), and Pao-Chu Hun (veterinarian, Laboratory Animal Center, KMU) for their excellent technical assistance.

## Conflict of interest

The authors declare that the research was conducted in the absence of any commercial or financial relationships that could be construed as a potential conflict of interest.

## Publisher’s note

All claims expressed in this article are solely those of the authors and do not necessarily represent those of their affiliated organizations, or those of the publisher, the editors and the reviewers. Any product that may be evaluated in this article, or claim that may be made by its manufacturer, is not guaranteed or endorsed by the publisher.
